# The effectiveness of care robots in alleviating physical burden and pain for caregivers: Non-randomized prospective interventional study – Preliminary study

**DOI:** 10.1097/MD.0000000000040877

**Published:** 2024-12-13

**Authors:** Jae Ik Jung, Yoo Seok Jeong, Dong Rak Kwon

**Affiliations:** a Department of Rehabilitation Medicine, School of Medicine, Daegu Catholic University, Daegu, Republic of Korea; b BioHealth Convergence Center, Daegu Technopark, Daegu, Republic of Korea.

**Keywords:** care robot, caregiver, muscle activity, pain, physical burden

## Abstract

**Background:**

Caregiver burden significantly affects both patients and caregivers but is often overlooked in clinical practice. Physical and emotional strain on caregivers can compromise the quality of care. Care robots are emerging as solutions to alleviate these burdens by assisting with routine tasks, thereby reducing caregivers’ physical strain and stress. Therefore, a prospective, non-randomized, interventional clinical trial was designed to identify changes in caregivers’ physical activities due to care robot use and explore the associated benefits.

**Methods:**

Twenty-two caregivers (1 male and 21 female; mean age, 62.05 years) were participated. We analyzed the impact of care robots on reducing physical burden and assessed caregivers’ satisfaction with these robots by examining care activity history, pain levels, muscle activities, and data for each physical care activity (e.g., transfer, reposition, feeding, and toileting), categorizing them based on whether a care robot was used. Care robots that assist in transfer, repositioning, feeding, and toileting activities were used in a clinical trial. Muscle activity was measured during maximum voluntary isometric contraction using electromyography sensors placed on the upper trapezius, biceps brachii, and erector spinae muscles.

**Results:**

During care robot use, we observed a statistically significant decrease in the distance and number of steps required for assisting with repositioning. However, the caregiving activity time increased when the robots assisted with transfer, feeding, and toileting (*P* < .001). Pain was significantly reduced during transfer and toileting activities using the care robot (*P* < .001) as well as during feeding activity (*P* = .040). Maximum voluntary isometric contraction showed a significant reduction in the upper trapezius, biceps brachii, and erector spinae muscles during the 4 care activities when using the robot, and these findings were consistent across the 3 sessions (*P* < .001). In the survey, caregivers indicated that “transfer-assisting” care robots were the most needed (15 respondents, 68.2%).

**Conclusion:**

In this study, we demonstrated that employing care robots can aid in mitigating muscle overuse among caregivers, potentially alleviating pain due to musculoskeletal conditions.

## 1. Introduction

Providing affordable and high-quality care for older adults and individuals requiring assistance due to their inability to perform activities of daily living remains a top priority for developed nations, especially for those experiencing demographic shifts due to declining birth rates and aging populations. As one of the most rapidly aging countries in the world, Korea is expected to become a super-aged society by 2025. The proportion of the population aged 65 years and above increased from 7% in 1999 to 11.8% in 2012 and 14% in 2017, and is projected to reach 20.8% by 2026.^[1]^ According to the World Population Prospects 2022 report from the United Nations, the global proportion of individuals aged 65 years and above is increasing faster than that of individuals below that age. Moreover, the global percentage of this population is expected to increase from 10% in 2022 to 16% in 2050.^[2]^

Consequently, securing an adequate number of skilled caregivers has become difficult owing to the diminishing working-age population. By 2030, 750,000 formal caregivers are projected to be needed, but this number is expected to fall by 110,000.^[3]^ This phenomenon is expected to increase the workload of available caregivers and yield negative consequences, including stress, musculoskeletal disorders, and poor quality of life.^[4–6]^

Addressing the challenge of providing adequate aid to care recipients while alleviating the physical burden on caregivers can be achieved by adopting care robots.^[7,8]^ Care robots are partially or fully autonomous machines that perform care-related activities for people with physical and/or mental handicaps related to age and/or health restrictions.^[9]^ These robots have been developed to provide physical support to caregivers in various care activities including transfer, repositioning, feeding, toileting, and bathing.^[10–12]^ Care robots are expected to provide high-quality care by reducing physical strain and stress on caregivers.

Research on the feasibility, usability, and safety of care robots for people with disabilities and older adults with mobility disorders has focused on care recipients.^[13,14]^ Caregiver burdens affect both the patient and the caregiver but are often overlooked by clinicians. Studies on the effectiveness of care robots regarding the workload, physical burden, and pain of caregivers owing to caregiving activities are insufficient.

To investigate the benefits of care robots for caregivers, we employed a novel application (BioHealth Convergence Center, Daegu Technopark, Republic of Korea) capable of collecting comprehensive information about the physical activities involved in caregiving. This application was developed at the BioHealth Convergence Center, Daegu Technopark, Republic of Korea, and its feasibility was confirmed in a pilot study.^[15]^

This clinical study aimed to identify changes in caregivers’ physical activities resulting from the use of care robots and explore the value and benefits of their use.

## 2. Materials and methods

### 2.1. Participants

In this prospective clinical trial, we evaluated the physical activity data of 22 caregivers working at a long-term nursing care facility for older adults and 2 residential homes for people with severe disabilities. The participants were enrolled between March 2022 and July 2022. Participants were included based on the following criteria: caregivers who worked at least 4 days weekly, had no communication difficulties, could comprehend questions, and had no history of musculoskeletal disorders before working as caregivers. Exclusion criteria were caregivers with central nervous system disorders, peripheral neuropathy, neuromuscular disorders, history of brain/spinal or other types of surgery, joint contracture, hypersensitivity to surface electrodes, dementia, cognitive dysfunction, alcohol or drug abuse, and an inability to communicate in Korean. The study was approved by the Institutional Review Board (IRB) and Independent Ethics Committee of the University Medical Center on December 29, 2021 (IRB No. CR-21-185) and adhered to the Declaration of Helsinki guidelines. This clinical study is registered at https://cris.nih.go.kr (KCT0009427). Written informed consent was obtained from all the participants.

### 2.2. Clinical trial procedure

**Figure 1.**
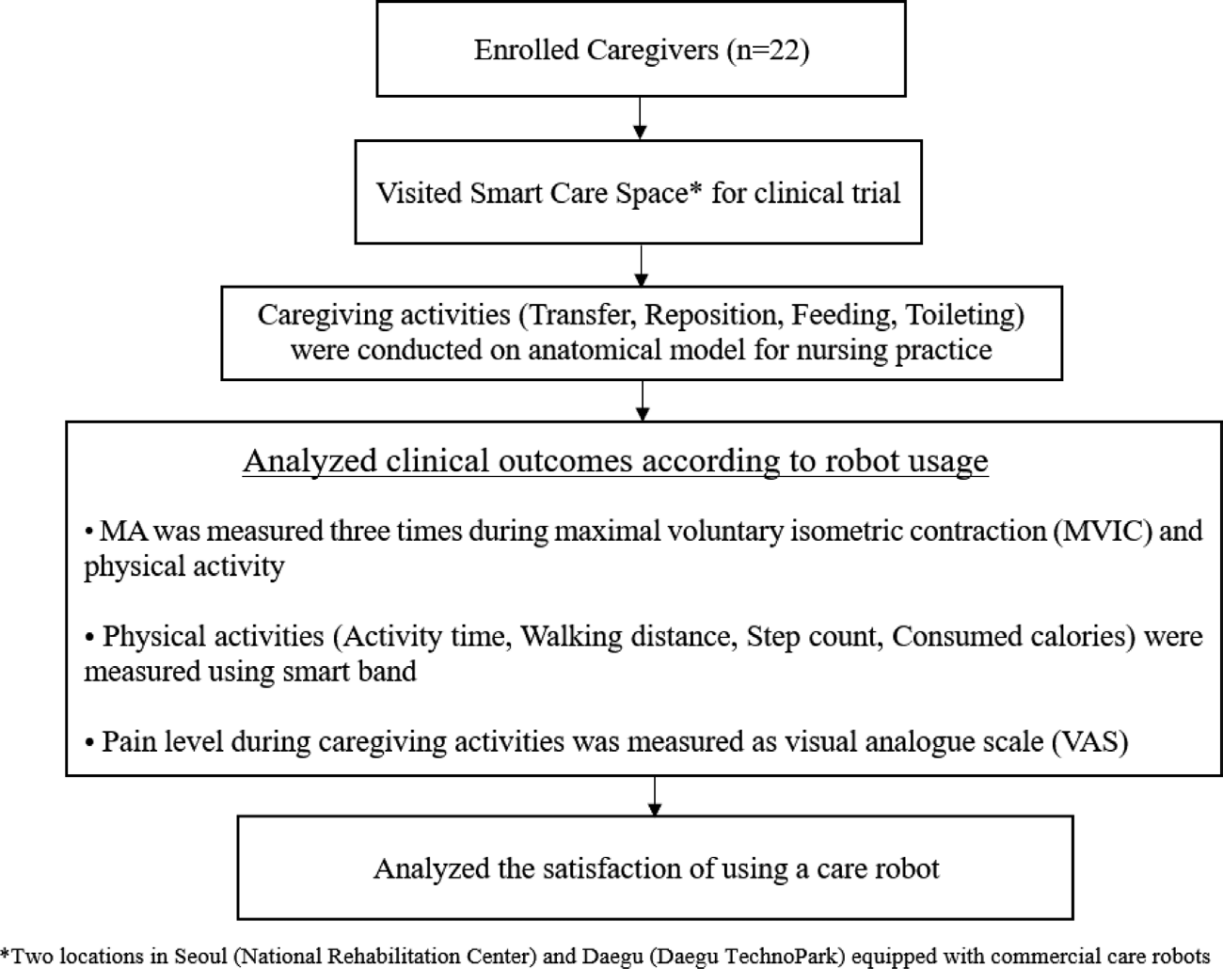
Study flowchart.

**Figure 2.**
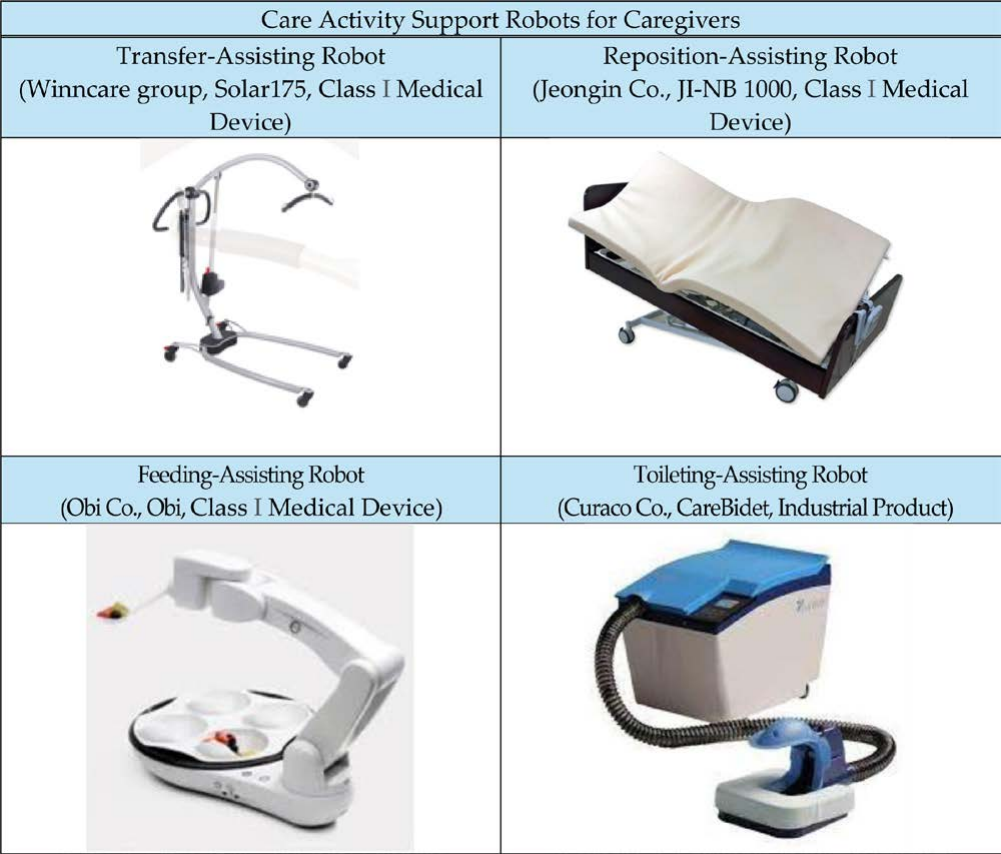
Types of care robots for caregivers.

Figure 1 illustrates a flowchart of the clinical trial. All caregivers volunteered to participate after comprehending the purpose and procedures of the study. A non-randomized prospective intervention study was adopted for the study design. The research was conducted in a Smart Care Space where clinical trials were conducted. The Smart Care Space denotes 2 sites in Seoul (National Rehabilitation Center) and Daegu (Daegu Technopark) equipped with commercial care robots that assist with transfer, repositioning, feeding, and toileting (Fig. 2). These spaces serve as simulated environments for caregiving activities in the home and care facility settings. During clinical trials, caregivers used a mobile application (BioHealth Convergence Center, Daegu Technopark, Republic of Korea) and a connected wearable smart band (Mi Band 1A, Xiaomi Inc., Beijing, China) that automatically logged their care activities and physical activity/burden. We analyzed the impact of care robots on reducing physical burden and evaluated caregivers’ satisfaction with these robots by assessing the care activity history, musculoskeletal pain levels, muscle activities (MAs), and physical activity data, categorizing them based on whether a care robot was used for each care activity (e.g., transfer, reposition, feeding, and toileting). Caregiving activities were conducted using human models due to the impact of COVID-19. Instead of directly applying them to patients, we applied them to a human model resembling a patient. The caregiving scenarios varied depending on whether care robots were employed. All caregiving activities were completed during the first visit to the Smart Care Space, and all clinical outcomes were measured.

### 2.3. Care robots

In this clinical trial, caregivers were instructed to use care robots that assisted with transfer, repositioning, feeding, and toileting (see Fig. 2). Solar 175 (Winncare Group Co., United Kingdom): This is a transfer-assisting robot that consists of an electric mobile lift. Its design, along with the lifting arm, provides ample space and comfort for users during transfers, facilitating easy movement of individuals who are unable to move independently. JI-NB 1000 (Jeongin Co., South Korea): this repositioning assistance robot is designed for automatic position changes in beds for people with disabilities, patients, and the elderly. It allows the upper body or legs to be raised to the desired angle and enables side-to-side repositioning. Obi (Obi Co., Germany): this adaptive feeding robot is intended for individuals with limitations in upper-extremity strength and mobility. This empowers users to control what and when they eat, thereby enhancing their autonomy during mealtimes. Care Bidet (Curaco Co., South Korea): this nursing care bidet functions as an automatic toileting aid system for individuals with limited mobility. Equipped with advanced built-in sensors, it detects urine and feces, automatically flushes excrement, rinses, and air-dries the user’s body, ensuring cleanliness and comfort without the need for disposable paper diapers.

## 3. Outcome measurements

### 3.1. Physical activities in caregivers

Throughout the study, caregivers’ physical activity was evaluated using a wearable smart band (Mi Band 1A, Xiaomi Inc., Beijing, China). Data analysis was conducted based on the specific caregiving activities undertaken. Activity time and level (walking distance, step count, and energy (calorie) expenditure) were assessed based on the caregiving tasks performed and whether robots were utilized during these activities.

### 3.2. Muscle activity in caregivers

A commercial Internet of Things, based electromyography (EMG) device (Delsys Trigno Wireless EMG System, Delsys Inc., Boston, MA) was utilized.^[16]^ This device was equipped with EMG and 6-axis sensors for measuring body movement and connected to a mobile application (BioHealth Convergence Center, Daegu Technopark, Republic of Korea). Surface EMG signals were collected using Trigno Standard sensors (Delsys, Boston, MA). These sensors utilize silver-contact wireless bipolar bar electrodes with fixed 10 mm interelectrode spacing. They are equipped with motion artifact suppression (patents) capabilities and can be moved freely. The sensors transmit data wirelessly and offer a 16-bit resolution, 20 to 450 Hz bandwidth, and baseline noise level of 750 nV. To obtain average data for various caregiving activities, each activity was repeated 3 times for analysis. Additionally, to evaluate the changes in MA, maximum voluntary isometric contractions (MVICs) were measured before app usage. MVIC was measured through manual muscle examination to normalize the EMG signals from the upper trapezius, biceps brachii, and erector spinae muscles. The mean values (mV) obtained from the EMG device for these muscles were divided by the corresponding MVIC values to yield normalized values (%MVIC). Data analyses were then conducted using %MVIC values. The collected data were analyzed using EMG software (EMGworks Analysis-Delsys, Boston, MA) with a 1 kHz sampling rate. To eliminate noise, a 20-Hz low-pass filter and a 500-Hz high-pass filter were applied. Muscle signals were analyzed using the root mean square. The surface EMG sensor was positioned on the upper trapezius muscle midway between the C7 spinous process and acromion lateral border. For the upper trapezius assessment, manual resistance was applied to the arm using a custom-made stabilizer and rope, while the shoulder was elevated in a standing position. For the biceps brachii muscle, a surface EMG sensor was positioned at the mid-arm level. Biceps brachii MVIC strength was measured at 90° elbow flexion on a customized bench with a rope. The sensor for the erector spinae muscle was attached laterally to the L1/L2 spinous processes. The participants’ lower legs were secured, and they wore a chest harness to resist trunk extension while lying prone on the table. A blinded physiatrist provided verbal encouragement and visual feedback, guiding the participants to exert maximum effort. Each participant completed 3, 5-second MVICs for the shoulder, arm, and back, with a 60-second rest between attempts. Values recorded during the middle 3 seconds were used for the data analysis. MA was measured 3 times within 1 day during caregiving activities. %MVIC was measured using electromyography sensors at the upper trapezius, biceps brachii, and erector spinae muscles.

### 3.3. Caregivers’ subjective perception of musculoskeletal pain level

To evaluate the subjective experience of musculoskeletal symptoms resulting from caregiving activities, we employed a visual analog scale (VAS) ranging from 0 to 10 points. This scale was specifically used to measure musculoskeletal pain in designated areas including the neck, shoulder, arm/elbow, hand/wrist, back, and leg. Participants were instructed to rate their pain levels in each area concerning 4 caregiving tasks (transferring, repositioning, feeding, and toileting) and their correlation with the use of robots. Additionally, Figure S1, Supplemental Digital Content, http://links.lww.com/MD/O155 illustrates the location and intensity of pain.

### 3.4. Clinical Trial Participant Questionnaire

The Clinical Trial Participant Questionnaire was developed specifically for this study, with careful consideration of process and usability factors. Although not an established standardized tool, we designed questionnaire items with reference to IEC 62366-1 standards on the usability of medical devices. Before commencing the clinical trials, participants completed a survey to evaluate their caregiving activity status, factors contributing to physical burden, and their demand for care robots. Upon completion of the clinical trial, another survey was conducted among the participants to evaluate their satisfaction and identify areas for improvement in using care robots based on their caregiving activity experiences.

## 4. Statistical analysis

The G* Power program (ver. 3.1.9.7; Heinrich-Heine-Universität Düsseldorf, Düsseldorf, Germany) to calculate the sample size. The primary efficacy endpoint for the target population was the pain scale, with reference values from a previous study,^[17]^ which ranged from a pretreatment mean of 51.1 (SD = 17.8) to a posttreatment mean of 33.8 (SD = 21.6). With a significant level of 0.05 and a power of 95%, the required number of participants was 20. Considering a dropout rate of 10%, the calculated number of participants was 22. We used a paired *t* test to compare activity time and activity level metrics, including walking distance, step count, and consumed calories, across various caregiving activities and based on whether robots were utilized during these tasks. This statistical method was employed to determine whether the mean difference between paired observations was statistically significant. Using 2 sets of measurements (activity time and activity level) obtained from the same subjects under different conditions (with or without the use of care robots), we could compare measurements taken on the same individuals before and after the intervention (i.e., using care robots).

A Generalized Linear Model was utilized to compare activity time, walking distance, step count, and calories consumed based on caregiving activities, the use of robots, and the degree of usage. This model was also used to assess differences in pain and MA (%MVIC) before and after using care robots for caregiving activities as well as by specific body regions. Correlation analyses were conducted using Pearson coefficients. Statistical analysis was conducted using the IBM SPSS software package for Windows (version 19.0, Chicago, IL), with statistical significance set at *P* < .05.

## 5. Results

### 5.1. Participant characteristics

This study included 1 male and 21 female participants, with a mean age of 62.05 ± 6.27 years, mean height of 159.27 ± 4.25 cm, and mean weight of 59.18 ± 7.47 kg. Among the participants, 19 were nursing assistants, 1 was a rehabilitation therapist, and 2 were activity assistants. The mean period of caregiving work experience was 128.68 ± 67.85 months (Table 1).

**Table 1:** Characteristics of participants.

Characteristics	Value
Age (years)	62.05 ± 6.27
Sex	
Male	1 (4.5%)
Female	21 (95.5%)
Occupation	
Nursing assistant	19 (86.4%)
Rehabilitation therapist	1 (4.5%)
Activity assistant	2 (9.1%)
Height (cm)	159.27 ± 4.25
Weight (kg)	59.18 ± 7.47
Period of work experience (months)	128.68 ± 67.85

Values are presented as mean ± standard deviation or number (%).

### 5.2. Physical activities in caregivers

Regarding transfer assistance, care robots significantly increased the activity time (Table 2). However, there was no statistically significant impact on activity levels (walking distance, step count, and calorie consumption). Regarding repositioning assistance, care robot use induced a statistically significant decrease in walking distance and step count. Furthermore, the activity time decreased with each subsequent session for transfer and toileting activities (see Table S1, Supplemental Digital Content, http://links.lww.com/MD/O156, which illustrates the clinical outcomes based on care activity, care robot usage, and sessions).

**Table 2:** Changes in clinical outcomes before and after using care robots.

Care activity	Outcome	Use of care robot		Difference	*P*-value
		Before	After		
Transfer	Activity time (minutes)	4.52 ± 0.52	14.65 ± 1.48	−10.14 ± 1.46	<.001*
	Walking distance (meter)	79.24 ± 27.00	88.03 ± 35.97	−8.79 ± 31.43	.204
	Step count (n)	110.00 ± 32.13	121.36 ± 47.51	−11.36 ± 41.91	.217
	Consumed calories (kcal)	2.56 ± 1.74	2.53 ± 1.67	0.03 ± 1.70	.934
Reposition	Activity time (minutes)	3.03 ± 0.29	3.02 ± 0.19	0.02 ± 0.32	.825
	Walking distance (meter)	10.45 ± 8.56	3.48 ± 10.81	6.97 ± 11.17	.008*
	Step count (n)	14.91 ± 12.06	5.08 ± 14.74	9.83 ± 15.30	.007*
	Consumed calories (kcal)	0.17 ± 0.25	0.08 ± 0.36	0.09 ± 0.33	.208
Feeding	Activity time (minutes)	4.02 ± 0.45	7.70 ± 0.81	−3.68 ± 0.85	<.001*
	Walking distance (meter)	1.36 ± 2.22	1.36 ± 2.45	0.00 ± 3.09	.999
	Step count (n)	2.12 ± 3.54	1.71 ± 3.05	0.41 ± 4.63	.683
	Consumed calories (kcal)	0.05 ± 0.16	–	0.05 ± 0.16	.186
Toileting	Activity time (minutes)	4.18 ± 0.69	10.03 ± 1.09	−5.85 ± 1.14	<.001*
	Walking distance (meter)	1.82 ± 2.46	3.33 ± 4.82	−1.52 ± 4.33	.116
	Step count (n)	3.00 ± 3.63	4.24 ± 6.79	−1.24 ± 6.46	.377
	Consumed calories (kcal)	–	0.03 ± 0.10	−0.03 ± 0.10	.162

Values are presented as mean ± standard deviation. –: All values are zero.

* *P* < .05 calculated using a paired *t* test before and after using care robots.

The caregiving activity time decreased when assisting with repositioning but increased when assisting with transfer, feeding, and toileting.

### 5.3. Muscle activities

No significant differences were observed among the MVIC (mV) values measured across the sessions (Table 3). However, the mean %MVIC of the shoulder muscle decreased from 22.98 ± 11.54 to 7.79 ± 5.35 for transfer activity using care robots. %MVIC was significantly reduced in the upper trapezius, biceps brachii, and erector spinae muscles for transfer, reposition, feeding, and toileting activities when using robots. These results were consistent throughout the sessions (*P* < .05) (Table 4).

**Table 3:** Maximal voluntary isometric contraction (mV) values of the 3 body parts measured across 3 sessions.

Body part	MVIC			*P*-value
	1 Session	2 Session	3 Session	
Shoulder	0.10 ± 0.06	0.12 ± 0.07	0.13 ± 0.06	.471
Arm	0.11 ± 0.05	0.12 ± 0.07	0.12 ± 0.06	.764
Back	0.04 ± 0.02	0.04 ± 0.01	0.04 ± 0.02	.996

Values were presented as mean ± standard deviation.

mV = mean values, MVIC = maximal voluntary isometric contraction.

*P* value calculated using a generalized linear model.

**Table 4:** Percentage of maximal voluntary isometric contraction of the 3 body parts based on care activity, care robot usage, and sessions.

Care activity	Body part	Session	Use of care robot		*P*-value		
			Before	After	Robot	Session	Robot × session
Transfer	Shoulder	1	21.82 ± 12.28	5.82 ± 3.29	<.001*	.338	.936
		2	23.18 ± 11.05	8.27 ± 5.53			
		3	23.95 ± 11.70	9.27 ± 6.38			
		Mean	22.98 ± 11.54	7.79 ± 5.35			
	Arm	1	32.50 ± 25.49	5.64 ± 3.75	<.001*	.852	.910
		2	32.41 ± 23.46	8.23 ± 5.43			
		3	33.14 ± 24.64	9.27 ± 6.71			
		Mean	32.68 ± 24.17	7.71 ± 5.57			
	Back	1	42.32 ± 16.06	10.23 ± 3.60	<.001*	.385	.973
		2	44.00 ± 15.06	12.77 ± 4.31			
		3	45.09 ± 14.92	14.05 ± 5.07			
		Mean	43.80 ± 15.16	12.35 ± 4.59			
Reposition	Shoulder	1	25.68 ± 12.96	9.32 ± 6.48	<.001*	.870	.819
		2	24.36 ± 11.86	10.27 ± 7.65			
		3	23.45 ± 11.00	9.45 ± 7.81			
		Mean	24.50 ± 11.82	9.68 ± 7.23			
	Arm	1	21.45 ± 15.41	16.55 ± 10.61	.043*	.930	.989
		2	20.82 ± 15.63	16.50 ± 11.18			
		3	20.50 ± 16.40	15.36 ± 9.89			
		Mean	20.92 ± 15.58	16.14 ± 10.42			
	Back	1	35.50 ± 12.55	28.05 ± 10.39	<.001*	.658	.983
		2	34.36 ± 11.64	27.73 ± 9.83			
		3	33.32 ± 10.90	26.09 ± 8.70			
		Mean	34.39 ± 11.57	27.29 ± 9.55			
Feeding	Shoulder	1	11.91 ± 7.78	5.09 ± 3.04	<.001*	.899	.984
		2	12.00 ± 8.02	4.73 ± 3.38			
		3	12.41 ± 7.48	5.45 ± 4.02			
		Mean	12.11 ± 7.65	5.09 ± 3.46			
	Arm	1	11.73 ± 8.85	4.91 ± 3.19	<.001*	.924	.918
		2	10.73 ± 8.04	4.82 ± 3.14			
		3	10.95 ± 8.35	5.18 ± 3.87			
		Mean	11.14 ± 8.30	4.97 ± 3.37			
	Back	1	27.77 ± 10.65	15.68 ± 7.18	<.001*	.802	.717
		2	27.41 ± 11.38	15.14 ± 6.09			
		3	25.27 ± 9.52	15.73 ± 6.18			
		Mean	26.82 ± 10.44	15.52 ± 6.41			
Toileting	Shoulder	1	29.64 ± 15.66	9.05 ± 5.79	<.001*	.310	.951
		2	31.55 ± 16.55	12.55 ± 7.70			
		3	33.14 ± 17.78	14.00 ± 9.81			
		Mean	31.44 ± 16.49	11.86 ± 8.09			
	Arm	1	27.09 ± 21.97	9.59 ± 5.69	<.001*	.658	.753
		2	26.32 ± 20.28	13.59 ± 8.53			
		3	28.18 ± 21.48	14.77 ± 9.41			
		Mean	27.20 ± 20.94	12.65 ± 8.22			
	Back	1	40.32 ± 11.48	16.73 ± 4.63	<.001*	.034	.512
		2	42.18 ± 12.90	22.73 ± 7.43			
		3	43.64 ± 14.14	24.68 ± 7.79			
		Mean	42.05 ± 12.76	21.38 ± 7.48			

Values were presented as mean ± standard deviation.

* *P* < .05 calculated using a generalized linear model.

For transfer, repositioning, and feeding assistance, a positive correlation was observed between MA (%MVIC) and activity time when robots were not used, but this was not statistically significant (Table 5). However, when using the transfer-assisting robot, a statistically significant negative correlation was observed between the MA (%MVIC) in the back area and activity time.

**Table 5:** Correlation between maximal voluntary isometric contraction values of the 3 body parts and activity time, walking distance, step count, and consumed calories in care activities based on care robot use.

Care activity	Use of care robot	Body part	Activity time	Walking distance	Step count	Consumed calories
Transfer	Before	Shoulder	0.00 (0.99)	0.04 (0.74)	0.06 (0.63)	−0.04 (0.75)
		Arm	0.08 (0.54)	−0.02 (0.88)	−0.03 (0.83)	0.01 (0.95)
		Back	0.07 (0.58)	0.30 (0.01*)	0.24 (0.05)	0.32 (0.01*)
	After	Shoulder	−0.11 (0.39)	0.13 (0.30)	0.16 (0.20)	0.03 (0.81)
		Arm	−0.22 (0.07)	0.30 (0.01*)	0.34 (0.01*)	0.18 (0.16)
		Back	−0.47 (<0.001*)	0.36 (<0.001*)	0.4 (<0.001*)	0.25 (0.04*)
Reposition	Before	Shoulder	0.01 (0.92)	0.16 (0.20)	0.15 (0.24)	0.17 (0.17)
		Arm	0.03 (0.81)	0.14 (0.26)	0.11 (0.39)	0.23 (0.06)
		Back	0.03 (0.78)	0.21 (0.10)	0.20 (0.11)	0.24 (0.05)
	After	Shoulder	0.27 (0.03*)	−0.08 (0.52)	−0.09 (0.48)	−0.05 (0.71)
		Arm	0.21 (0.09)	−0.14 (0.26)	−0.15 (0.23)	−0.15 (0.24)
		Back	0.00 (0.97)	0.22 (0.08)	0.24 (0.05)	0.17 (0.18)
Feeding	Before	Shoulder	0.19 (0.12)	−0.10 (0.41)	0.16 (0.20)	−0.08 (0.54)
		Arm	0.05 (0.68)	−0.13 (0.31)	0.34 (0.01*)	−0.14 (0.27)
		Back	0.17 (0.17)	−0.11 (0.37)	0.4 (<0.001*)	−0.07 (0.59)
	After	Shoulder	0.34 (0.01*)	−0.02 (0.89)	0.17 (0.05)	–
		Arm	0.04 (0.75)	0.04 (0.74)	0.03 (0.70)	–
		Back	−0.32 (0.01*)	−0.10 (0.41)	0.27 (<0.001*)	–
Toileting	Before	Shoulder	−0.04 (0.75)	−0.13 (0.29)	−0.10 (0.40)	–
		Arm	−0.20 (0.10)	−0.15 (0.24)	−0.09 (0.46)	–
		Back	−0.02 (0.86)	0.05 (0.71)	0.07 (0.58)	–
	After	Shoulder	−0.25 (0.04*)	0.05 (0.70)	−0.05 (0.71)	−0.06 (0.61)
		Arm	−0.12 (0.34)	0.02 (0.87)	−0.09 (0.48)	−0.07 (0.59)
		Back	−0.53 (<0.001*)	−0.03 (0.81)	0.05 (0.69)	0.00 (0.98)

Values are presented as correlation coefficients (*P*-value). –: All values are zero.

* *P* < .05 calculated using Pearson correlation analysis.

### 5.4. Musculoskeletal pain level

We assessed the body regions where the participants experienced the most intense pain and their corresponding self-reported pain intensity (measured using the VAS). This assessment was based on each participant’s caregiving activities and robot usage (see Table S2, Supplemental Digital Content, http://links.lww.com/MD/O156, which illustrates the location of pain and VAS for caregivers perceived highest based on care activity and robot usage). The number of participants reporting pain decreased from 22 during transfer, repositioning, feeding, and toileting activities to 18, 1, 9, and 14, respectively, upon the introduction of the robots. Pain was significantly reduced for each activity using care robots (*P* < .05, Table 6).

**Table 6:** Changes in pain level before and after using care robots.

Care activity	Outcome	Use of care robot		Difference	*P*-value
		Before	After		
Transfer	VAS (n = 18)	2.36 ± 0.55	1.32 ± 0.45	1.03 ± 0.59	<.001*
Reposition	VAS (n = 1)	–	–	–	–
Feeding	VAS (n = 9)	1.78 ± 0.61	1.11 ± 0.33	0.67 ± 0.82	.040*
Toileting	VAS (n = 14)	2.29 ± 0.45	1.24 ± 0.42	1.04 ± 0.52	<.001*

Values are presented as means ± standard deviation. –: All values are zero.

VAS = visual analogue scale.

* *P* < .05 calculated using a paired *t* test before and after using care robots.

The “Reposition” caregiving activity was excluded when comparing the results, as only 1 participant reported pain when using care robots. Nonetheless, the number of individuals experiencing pain decreased from 22 to 1 when robots were used under the same conditions.

Using care robots for transfer and toileting assistance resulted in a statistically significant decrease in pain during these activities and in different body areas (see Table S3, Supplemental Digital Content, http://links.lww.com/MD/O156, which illustrates the VAS based on location according to care activity and robot usage). Transfer activities tend to cause increased pain in body areas 6, 9, 17, 21, and 23 compared with other body areas.

### 5.5. Questionnaire outcomes before clinical trials

Among the 22 participants, 14 (63.6%) identified “transfer assistance” as the caregiving task most significantly associated with the occurrence of illness and stress during caregiving activities. “Reposition assistance” was selected by 5 participants (22.7%), followed by “toileting assistance” (n = 2, 9.1%) and “feeding assistance” (n = 1, 4.5%). These findings suggest that transfer assistance is considered the most demanding caregiving task among the participants.

Responses to a multiple-choice question regarding the primary causes of physical burden during caregiving activities revealed “psychological stress” to be the most common factor, with 15 individuals (34.9%) selecting it. This was followed by “overworking” (n = 11, 25.6%), “lack of sleep” (n = 8, 18.6%), “irregular working time” (n = 6, 14.0%), and “excessive work schedules” (n = 3, 7.0%). These responses highlighted the various factors contributing to the physical burden among caregivers, with psychological stress being the most prevalent concern.

When prioritizing their needs and selecting the most effective caregiving robot for alleviating physical burden and pain, 18 participants (81.8%) chose “transfer-assisting robots” as their top choice. “Toileting-assisting robots” were selected by 2 participants (9.1%), while “feeding-assisting robots” and “reposition-assisting robots” were each chosen by 1 respondent (4.5%). These preferences indicate that caregivers prioritize transfer assistance when considering the potential benefits of care robots.

### 5.6. Questionnaire outcomes after clinical trials

Regarding the effectiveness of transfer-assisting robots, 14 participants (63.6%) expressed their satisfaction by answering “satisfied.” Four participants (18.2%) answered “highly satisfied” and “neither satisfied nor dissatisfied,” indicating that none of them were dissatisfied. Regarding the improvements needed for this robot, 7 participants (31.8%) answered that “safety in use” was the key area for improvement. This was followed by “complexity of use” and “none,” chosen by 5 participants each (22.7%), while “convenience of use” was selected by 3 participants (13.6%).

Participants showed predominantly positive responses, with 86.4% expressing satisfaction with the effectiveness of the reposition-assisting care robots. Thirteen participants (59.1%) stated that no improvement was required. Four participants (18.2%) answered “practicality of use,” while “etc” was selected by 3 participants (13.6%). Additionally, 7 (31.8%) and 9 (40.9%) participants reported satisfaction with the feeding-assisting and toileting-assisting robots, respectively, a less positive response compared to the transfer- and reposition-assisting robots.

Regarding which care robots were most expected to undergo rapid development and adoption for alleviating physical fatigue, 15 participants (68.2%) selected “transfer-assisting robots.” This was followed by “reposition-assisting robots” (59.1%), “toileting-assisting robots” (63.6%), and “feeding-assisting robots” (72.7%).

Regarding which entities participants believed should lead the development, dissemination, and policy of caregiving technology, with the option for multiple responses, 16 participants (36.4%) selected “Government” as the most important entity. “Research institutes’ were selected by 10 participants (22.7%), while “Local government” and “Policy/Regulatory agencies” were each selected by 4 participants (9.1%).

## 6. Discussion

We compared robot-assisted and manual care regarding caregiver burden using measures such as MA and subjective ratings of perceived physical discomfort. Our findings revealed that care robots significantly reduced caregivers’ pain during transfer, feeding, and toileting activities. Regarding repositioning assistance, only one participant reported pain among the 22 caregivers using the robot. Hence, the use of care robots is highly beneficial for preventing and managing musculoskeletal disorders among caregivers. Furthermore, incorporating ICT technology into the Smart Care Space facilitated the collection and analysis of data to assess the effects of care robots in alleviating physical burdens and pain relief among caregivers.

We observed that when care robots were used for repositioning assistance, there was a statistically significant decrease in the walking distance and step count. Additionally, caregiving activity time decreased when assisting with repositioning. However, there was an increase in the caregiving activity time with assistance for transfer, feeding, and toileting activities, which appeared to be correlated with the operational time of the care robots. This observation highlights the need to develop robots with shorter operation times and enhanced user-friendliness. Additionally, because caregivers only used robots for a single day, they were initially unfamiliar with their operations. However, the activity time significantly decreased in each session, indicating that participants gained proficiency over time.

The mean %MVIC of the shoulder muscle decreased during the transfer activity when using care robots. %MVIC was significantly reduced in the upper trapezius, biceps brachii, and erector spinae muscles during transfer, reposition, feeding, and toileting caregiving activities when using the robots. These results remained consistent throughout the 3 sessions. Furthermore, caregivers reported more frequent and higher pain levels in the lower back than in other body areas during transfer activities. The lower back also exhibited the highest percentage of MVIC for all caregiving activities. This finding aligns with previous studies describing the musculoskeletal discomfort experienced by caregivers caring for adults with physical disabilities.^[18–20]^

Previous studies on the use of transfer-assistive devices have demonstrated decreases in biomechanical stress, lower back pain, injury rates, and worker compensation costs when transfer-assistive robots are used.^[21,22]^ Previous study by Ko et al^[23]^ reported that using toileting-assisting robots was more effective in alleviating physical burden, as measured by pain (VAS) and MA (%MVIC), compared to manual toileting assistance activity.

In a previous study conducted by Mitzner et al,^[24]^ healthcare providers’ preferences and acceptance of assistance (human or robotic) for various caregiving tasks were demonstrated. The participants predominantly expressed positive opinions about robots for simple activities requiring physical exertion (e.g., transfer and reposition). However, for tasks directly related to safety and involving fine dexterity (e.g., intravenous use and operation of infusion pump devices), participants preferred to rely on human assistance. In our study, the transfer-assisting robot emerged as the most desired option among the caregivers. Additionally, the questionnaire survey showed that caregivers identified safety as an area needing improvement in care robots, particularly transfer-assisting robots. To meet these demands, a safe transfer-assisting robot is currently being developed.

A narrative review by Ballestra et al^[25]^ emphasized the impact of outcome expectations in managing chronic low back pain in working-age patients. The study highlighted those higher expectations of recovery, combined with personalized training, realistic views on pain relief, good communication, and a desire for explanations, were associated with better recovery outcomes.^[26]^ Similarly, the use of care robots objectively reduced musculoskeletal pain and improved physical activity, fostering positive expectations among caregivers. Understanding both caregivers and care recipients’ expectations regarding care robots may provide insights into how these expectations shape the benefits they derive from using robots. Since musculoskeletal pain in caregivers often develops chronically rather than acutely, the long-term use of care robots, supported by continuous communication among patients, caregivers, and healthcare providers, appears essential. Regular follow-ups to assess caregiver satisfaction and identify areas for improvement can enhance the efficacy of care robots in alleviating caregivers’ physical burden. By addressing these concerns and incorporating feedback, positive expectations can be nurtured, leading to improved overall results. Therefore, future research should focus on measuring expectations before and after care robot use and exploring how these expectations affect the perceived benefits.

The induction of different types of expectations, whether positive or negative, through verbal suggestions does not seem to influence the perception of acute pain during potentially discomfort-inducing procedures.^[27]^ However, a recent narrative review showed that positive verbal suggestions can have beneficial effects on chronic conditions such as osteoarthritis and low back pain, suggesting that verbal suggestions may play a role in modulating pain perception in long-term conditions.^[28]^ Despite these findings, no study has specifically examined whether verbal suggestions or induced expectations affect the experience of using care robots. This gap in the literature presents an opportunity to investigate whether verbal cues and expectations shape caregivers’ and care recipients’ experiences with care robots, potentially influencing their satisfaction, acceptance, and perceived benefits.

As caregiver satisfaction with the use of care robots was surveyed in our study, further research should explore the satisfaction of care recipients with care robots, which appears to depend on the type of disease and its chronicity. Patients with chronic conditions may have unmet expectations, leading to lower satisfaction despite improvements in clinical outcomes such as ulcer treatment.^[29]^ Additionally, patients’ needs for robotic assistance vary based on their functional disability, with customization being key for effectiveness.^[30]^ Robot acceptability depends on its perceived usefulness, with older individuals more likely to accept assistive devices that help maintain their independence when they see a clear need for them.^[31]^ In person-centered care, it is important to gather and respect personal information, experiences, and choices. Understanding these factors is important for creating care robots that meet the various needs of different patient groups.

Many participants have highlighted the complexity and practicality of care robots. Care robots with high usability must be introduced to the market to effectively bridge the gap between the product and the user, as well as to connect technological innovation with the environment and social systems supported by organizational innovation.^[32]^ In our study, caregivers identified the government as being primarily responsible for developing and disseminating care robots. Consistent investment and research into care robots led by governments will contribute to the realization of these technologies.

A previous study by Kim et al^[33]^ identified the social values of care robots using analytic hierarchy processes. The results showed that labor and health-related values were of the highest importance. Regarding labor value, care robots can assist caregivers by undertaking simple, repetitive tasks that typically impose physical burdens or stress. Caregiver burdens may negatively affect patient treatment and increase the risk of hospitalization and mortality.^[34,35]^ Therefore, simple and repetitive tasks performed by care robots should be considered when developing care services or when introducing new functions for care robots. This approach helps to uphold and maintain both labor and health-related values.

Care robots have the potential to greatly improve both the quality of care and well-being of caregivers and patients. By assisting with tasks such as transfers, repositioning, feeding, and toileting, they reduce the physical strain on caregivers, lower the risk of injury, and extend caregiver careers. This also helps to address caregiver shortages by allowing care robots to handle routine tasks, freeing caregivers to focus on more personalized care. However, the successful use of care robots requires proper training and user acceptance and satisfaction. Customization and ongoing feedback are key to making care robots truly effective in the clinical setting.

This study provides valuable insights with objective data on reduced physical strain, caregiver feedback, and surveys assessing caregiver satisfaction with care robots, which offers guidance for their future development and strategic implementation in clinical settings. Specifically, care robots can be integrated into care plans for patients with chronic conditions to reduce caregiver burnout and physical strain, and their customization based on patient-specific needs can improve outcomes in rehabilitation or long-term care facilities. These findings offer foundational data that can promote broader adoption of care robots in clinical practice, ultimately enhancing patient and caregiver outcomes.

## 7. Limitations and strength

Our study had several limitations. First, the sample size was relatively small, indicating the need for further large-scale studies to validate and generalize the findings. Second, because the experiments were conducted over a single day due to the COVID-19 pandemic, conducting prolonged clinical trials with real patients was not feasible. Therefore, further research is warranted to evaluate the long-term benefits of introducing care robots for caregivers. Third, care activities were conducted using human models for nursing practice rather than on actual care recipients. This might have distorted the results related to MA and subjective physical burden measured during care activities. Therefore, future research involving individuals requiring care is necessary to provide more accurate and reliable insights. Finally, we did not contemplate user feedback to explore the potential impact of care robots on job satisfaction and caregivers’ mental health despite “psychological stress, which emerged as the most common factor. Therefore, further studies are necessary to gather feedback on the impact of care robots on job contentment and psychological well-being of nursing personnel.

While our study acknowledges certain limitations, it also has notable strengths. Unlike many previous studies that primarily focused on the functionality of care robots and their impact on care recipients, our research emphasizes the value of care robots for caregivers. We objectively and quantitatively confirmed that the physical burden on caregivers was reduced by using care robots, as evidenced by the %MVIC measurements obtained using an EMG device.

## 8. Conclusions

In this study, we demonstrated that using care robots could effectively mitigate muscle overuse in caregivers, thereby alleviating the pain associated with musculoskeletal conditions. Furthermore, this study provides valuable clinical data for optimizing pain management strategies among caregivers. This study presents objective data on reduced physical strain, caregiver feedback, and surveys that assess caregivers’ satisfaction with care robots. This information is crucial for future development and strategic implementation of care robots in clinical settings. Furthermore, it provides foundational research data important for promoting the dissemination and adoption of care robots, ultimately enhancing the quality of care for both caregivers and patients. Finally, the study underscores the necessity for interventions involving care robots to reduce the physical burdens associated with caregiving.

## Acknowledgments

We thank all the patients who participated in this study and the investigators. We thank Sang Gyu Kwak PhD at Daegu Catholic University of Medicine for providing statistical guidance.

## Author contributions

**Conceptualization:** Yoo Seok Jeong, Dong Rak Kwon.

**Data curation:** Jae Ik Jung, Yoo Seok Jeong, Dong Rak Kwon.

**Investigation:** Yoo Seok Jeong, Dong Rak Kwon.

**Methodology:** Yoo Seok Jeong, Dong Rak Kwon.

**Project administration:** Yoo Seok Jeong, Dong Rak Kwon.

**Software:** Yoo Seok Jeong.

**Supervision:** Jae Ik Jung, Dong Rak Kwon.

**Validation:** Jae Ik Jung, Dong Rak Kwon.

**Visualization:** Jae Ik Jung, Dong Rak Kwon.

**Writing – original draft:** Jae Ik Jung, Dong Rak Kwon.

**Writing – review & editing:** Jae Ik Jung, Yoo Seok Jeong, Dong Rak Kwon.

## Supplementary Material


