# Supplementary material for: The effectiveness of care robots in alleviating physical burden and pain for caregivers: Non-randomized prospective interventional study – Preliminary study
Source: Medicine (Baltimore). 2024 Dec 13;103(50):e40877. doi: 10.1097/MD.0000000000040877 (PMC11651516; doi:10.1097/MD.0000000000040877)
Supplement: Supplementary file 1 [file medi-103-e40877-s001.docx]

**Supplemental Digital Content. Figure 1**.

| **※ Reference. Location and Intensity of Pain**   \| Location and Intensity of Pain \| \| \| \| --- \| --- \| --- \| \| Location of Pain \| \| Intensity of Pain (Visual analogue scale) \| \| 1. Anterior neck \| 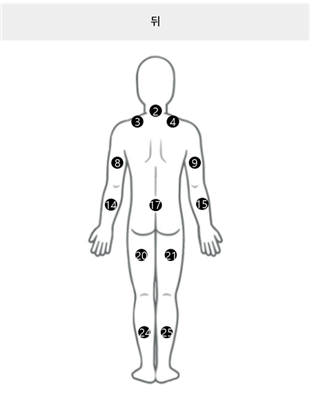  Back \| \| 0~1 \| No pain \| \| --- \| --- \| \| 2~3 \| Mild \| \| 4~5 \| Moderate \| \| 6~7 \| Severe \| \| 8~9 \| Very Severe \| \| 10 \| Worst Possible \|   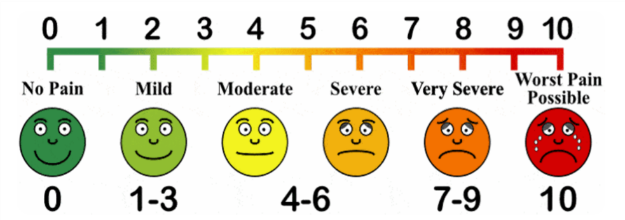 \| \| 2. Posterior neck \| \| 3. Upper trapezius (L) \| \| 4. Upper trapezius (R) \| \| 5. Pectoralis \| \| 6. Biceps (L) \| \| 7. Biceps (R) \| \| 8. Triceps (L) \| \| 9. Triceps (R) \| \| 10. Brachialis (L) \| \| 11. Brachialis (R) \| \| 12. Wrist (L) \| \| 13. Wrist (R) \| \| 14. Posterior forearm (L) \| \| 15. Posterior forearm (R) \| 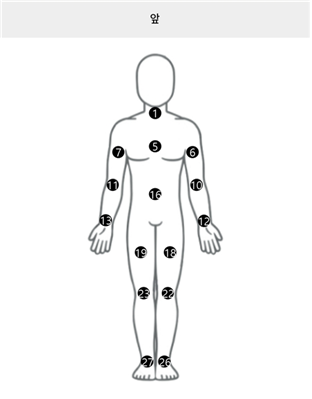  Front \|  \| \| 16. Abdominals \| \| 17. Erector spinae \| \| 18. Quadriceps (L) \| \| 19. Quadriceps (R) \| \| 20. Hamstrings (L) \| \| 21. Hamstrings (R) \| \| 22. Knee (L) \| \| 23. Knee (R) \| \| 24. Calf (L) \| \| 25. Calf (R) \| \| 26. Ankle (L) \| \| 27. Ankle (R) \| |
| --- | --- | --- | --- | --- | --- | --- | --- | --- | --- | --- | --- | --- | --- | --- | --- | --- | --- | --- | --- | --- | --- | --- | --- | --- | --- | --- | --- | --- | --- | --- | --- | --- | --- | --- | --- | --- | --- | --- | --- | --- | --- | --- | --- | --- | --- | --- | --- | --- | --- |
