# Supplementary material for: The effectiveness of care robots in alleviating physical burden and pain for caregivers: Non-randomized prospective interventional study – Preliminary study
Source: Medicine (Baltimore). 2024 Dec 13;103(50):e40877. doi: 10.1097/MD.0000000000040877 (PMC11651516; doi:10.1097/MD.0000000000040877)
Supplement: Supplementary file 2 [file medi-103-e40877-s002.docx]

**Supplemental Digital Content. Table 1.**

| Care Activity | Outcome | Session | Use of care robot | | *p*-value | | |
| --- | --- | --- | --- | --- | --- | --- | --- |
|  |  |  | Before After | | Robot | Session | Robot*Session |
| Transfer | Activity time(minutes) | 1 | 4.86 ± 0.83 | 16.55 ± 2.18 | <0.001* | <0.001 | <0.001* |
|  |  | 2 | 4.41 ± 0.59 | 14.27 ± 1.72 |  |  |  |
|  |  | 3 | 4.27 ± 0.55 | 13.14 ± 1.42 |  |  |  |
|  | Walking distance(meter) | 1 | 69.55 ± 37.6 | 85.45 ± 37.76 | 0.181 | 0.417 | 0.579 |
|  |  | 2 | 81.36 ± 34.82 | 92.27 ± 41.97 |  |  |  |
|  |  | 3 | 86.82 ± 26.97 | 86.36 ± 43.81 |  |  |  |
|  | Step count(n) | 1 | 97.68 ± 41.28 | 116.59 ± 48.79 | 0.175 | 0.346 | 0.570 |
|  |  | 2 | 110.91 ± 46.35 | 127.14 ± 55.70 |  |  |  |
|  |  | 3 | 121.41 ± 34.21 | 120.36 ± 56.64 |  |  |  |
|  | Consumed calories(kcal) | 1 | 2.00 ± 2.31 | 2.41 ± 1.94 | 0.932 | 0.372 | 0.637 |
|  |  | 2 | 2.68 ± 2.10 | 2.59 ± 2.11 |  |  |  |
|  |  | 3 | 3.00 ± 1.63 | 2.59 ± 2.04 |  |  |  |
| Reposition | Activity time(minutes) | 1 | 3.18 ± 0.39 | 3.09 ± 0.29 | 0.805 | 0.012 | 0.453 |
|  |  | 2 | 3.05 ± 0.38 | 3.00 ± 0.31 |  |  |  |
|  |  | 3 | 2.86 ± 0.47 | 2.95 ± 0.21 |  |  |  |
|  | Walking distance(meter) | 1 | 14.09 ± 19.43 | 0.91 ± 2.94 | 0.013* | 0.661 | 0.288 |
|  |  | 2 | 10.00 ± 13.09 | 6.36 ± 27.70 |  |  |  |
|  |  | 3 | 7.27 ± 10.32 | 3.18 ± 9.45 |  |  |  |
|  | Step count(n) | 1 | 19.95 ± 27.40 | 0.55 ± 2.56 | 0.011* | 0.919 | 0.203 |
|  |  | 2 | 12.68 ± 14.96 | 8.91 ± 38.37 |  |  |  |
|  |  | 3 | 12.09 ± 14.03 | 5.77 ± 14.94 |  |  |  |
|  | Consumed calories(kcal) | 1 | 0.23 ± 0.53 | - | 0.336 | 0.613 | 0.368 |
|  |  | 2 | 0.14 ± 0.47 | 0.23 ± 1.07 |  |  |  |
|  |  | 3 | 0.14 ± 0.35 | - |  |  |  |
| Feeding | Activity time(minutes) | 1 | 4.23 ± 0.61 | 8.32 ± 0.89 | <0.001* | <0.001* | 0.070 |
|  |  | 2 | 3.95 ± 0.49 | 7.59 ± 1.18 |  |  |  |
|  |  | 3 | 3.86 ± 0.56 | 7.18 ± 0.73 |  |  |  |
|  | Walking distance(meter) | 1 | 1.82 ± 5.01 | 1.82 ± 5.01 | 0.999 | 0.114 | 0.890 |
|  |  | 2 | 0.45 ± 2.13 | - |  |  |  |
|  |  | 3 | 1.82 ± 5.01 | 2.27 ± 6.12 |  |  |  |
|  | Step count(n) | 1 | 3.32 ± 9.01 | 2.41 ± 6.91 | 0.707 | 0.110 | 0.900 |
|  |  | 2 | 0.59 ± 2.77 | - |  |  |  |
|  |  | 3 | 2.45 ± 6.93 | 2.73 ± 7.03 |  |  |  |
|  | Consumed calories(kcal) | 1 | 0.09 ± 0.43 | - | 0.182 | 0.550 | 0.550 |
|  |  | 2 | - | - |  |  |  |
|  |  | 3 | 0.05 ± 0.21 | - |  |  |  |
| Toileting | Activity time(minutes) | 1 | 4.73 ± 0.88 | 12.18 ± 1.50 | <0.001* | <0.001* | <0.001* |
|  |  | 2 | 4.00 ± 0.69 | 9.50 ± 1.74 |  |  |  |
|  |  | 3 | 3.82 ± 0.73 | 8.41 ± 1.10 |  |  |  |
|  | Walking distance(meter) | 1 | 0.91 ± 2.94 | 5.00 ± 8.59 | 0.160 | 0.266 | 0.118 |
|  |  | 2 | 0.45 ± 2.13 | 2.27 ± 6.12 |  |  |  |
|  |  | 3 | 4.09 ± 7.34 | 2.73 ± 7.03 |  |  |  |
|  | Step count(n) | 1 | 2.73 ± 6.04 | 4.91 ± 8.41 | 0.383 | 0.140 | 0.492 |
|  |  | 2 | 0.45 ± 2.13 | 3.14 ± 9.16 |  |  |  |
|  |  | 3 | 5.82 ± 10.36 | 4.68 ± 9.80 |  |  |  |
|  | Consumed calories(kcal) | 1 | - | 0.05 ± 0.21 | 0.160 | 0.608 | 0.608 |
|  |  | 2 | - | 0.05 ± 0.21 |  |  |  |
|  |  | 3 | - | - |  |  |  |

Values are presented by mean ± standard deviation

^*^*P*<.05 calculated by generalized linear model

- : All values are zero

**Supplemental Digital Content. Table 2.**

| Care Activity | Caregiver | Robot used | | Robot not used | |
| --- | --- | --- | --- | --- | --- |
|  |  | Location | VAS | Location | VAS |
| Transfer | 1 | 4,17 | 2 | 10,11,12,13,6,7 | 1 |
|  | 2 | 23 | 3 |  |  |
|  | 3 | 17 | 4 | 12,13 | 2 |
|  | 4 | 17,27,4 | 2 | 27 | 1 |
|  | 5 | 17,4 | 3 | 4 | 1 |
|  | 6 | 17 | 2 | 17 | 1 |
|  | 7 | 17 | 3 | 13,7 | 1 |
|  | 8 | 11,12,3,6,9 | 3 | 6,7 | 1 |
|  | 9 | 17,4 | 3 | 4 | 2 |
|  | 10 | 17,3,4 | 3 | 4,7 | 2 |
|  | 11 | 17,6 | 3 | 17,4 | 1 |
|  | 12 | 12,13,17,3,4,8,9 | 2 |  |  |
|  | 13 | 6,7 | 3 |  |  |
|  | 14 | 12 | 3 | 12,13 | 1 |
|  | 15 | 17 | 3 | 10,11,3,4 | 1 |
|  | 16 | 13,17 | 3 | 4 | 2 |
|  | 17 | 12,21,4 | 3 |  |  |
|  | 18 | 17 | 3 | 12,13 | 1 |
|  | 19 | 12,13,17,3,4 | 2 | 13 | 2 |
|  | 20 | 17 | 3 | 13,4 | 2 |
|  | 21 | 12,13,3,4 | 3 | 3,4 | 2 |
|  | 22 | 12,13,17 | 2 | 17 | 1 |
| Reposition | 1 | 10 | 4 |  |  |
|  | 2 | 17 | 4 |  |  |
|  | 3 | 11 | 2 |  |  |
|  | 4 | 17,4 | 3 |  |  |
|  | 5 | 17,4 | 3 |  |  |
|  | 6 | 17 | 2 |  |  |
|  | 7 | 4 | 3 |  |  |
|  | 8 | 10,11,12,3,4,7 | 2 |  |  |
|  | 9 | 4 | 2 | 17 | 1 |
|  | 10 | 4 | 2 |  |  |
|  | 11 | 6,7 | 3 |  |  |
|  | 12 | 11,17,4,7 | 2 |  |  |
|  | 13 | 12,3 | 2 |  |  |
|  | 14 | 17 | 3 |  |  |
|  | 15 | 12,13,17,22 | 2 |  |  |
|  | 16 | 14,22,7 | 3 |  |  |
|  | 17 | 17 | 3 |  |  |
|  | 18 | 12,13 | 3 |  |  |
|  | 19 | 17,3,4 | 1 |  |  |
|  | 20 | 4 | 2 |  |  |
|  | 21 | 3 | 3 |  |  |
|  | 22 | 12,13,17 | 2 |  |  |
| Feeding | 1 | 4 | 1 | 17 | 2 |
|  | 2 | 22,23 | 3 | 22,23 | 1 |
|  | 3 |  |  | 26,27 | 2 |
|  | 4 | 4 | 4 | 27 | 1 |
|  | 5 | 11,13 | 2 |  |  |
|  | 6 | 17 | 1 |  |  |
|  | 7 | 17 | 3 | 23,27 | 1 |
|  | 8 | 11,13,4 | 2 | 4 | 1 |
|  | 9 | 4 | 2 | 13 | 1 |
|  | 10 | 4 | 2 |  |  |
|  | 11 | 17 | 2 |  |  |
|  | 12 | 11,13,4 | 2 |  |  |
|  | 13 | 17 | 3 |  |  |
|  | 14 | 17 | 1 | 17 | 1 |
|  | 15 | 12 | 2 | 18,18 | 1 |
|  | 16 | 13 | 2 |  |  |
|  | 17 | 4 | 3 |  |  |
|  | 18 | 17 | 1 | 17 | 1 |
|  | 19 | 17 | 1 |  |  |
|  | 20 | 13 | 2 |  |  |
|  | 21 |  |  |  |  |
|  | 22 | 17,23,26 | 1 |  |  |
| Toileting | 1 | 17 | 3 | 10,11,17 | 1 |
|  | 2 | 17 | 4 | 17 | 2 |
|  | 3 | 17 | 4 |  |  |
|  | 4 | 17 | 4 |  |  |
|  | 5 | 4 | 4 | 4,23 | 2 |
|  | 6 | 13,17 | 1 |  |  |
|  | 7 | 13,17,4 | 3 | 13,17 | 1 |
|  | 8 | 10,12,3,4,7,8 | 2 | 11 | 1 |
|  | 9 | 17,4 | 2 | 4,17 | 1 |
|  | 10 | 17,4 | 3 | 17 | 2 |
|  | 11 | 17 | 3 | 13,17 | 1 |
|  | 12 | 10,11,12,13,17,3,4 | 2 | 10,11 | 2 |
|  | 13 | 17 | 3 | 6 | 1 |
|  | 14 | 12 | 3 |  |  |
|  | 15 | 12,17,4 | 2 | 12,13,17 | 1 |
|  | 16 | 13 | 3 | 13 | 1 |
|  | 17 | 17,4 | 3 |  |  |
|  | 18 | 17 | 3 |  |  |
|  | 19 | 17,4 | 1 |  |  |
|  | 20 | 12,13,15,4 | 2 | 13 | 1 |
|  | 21 | 12,13,15,4,9 | 3 | 17 |  |
|  | 22 | 17 | 2 |  | 1 |

**Supplemental Digital Content. Table 3.**

| Care Activity | Location | Use of care robot | | *P* value | | | | |  |  |
| --- | --- | --- | --- | --- | --- | --- | --- | --- | --- | --- |
|  |  | Before After | | Robot | | Location | Robot*Location | |  |  |
| Transfer | 03 | 2.19 ± 0.53 | 1.25 ± 0.27 | <.001* | | 0.006* | <.001* | |  |  |
|  | 04 | 2.26 ± 0.49 | 1.48 ± 0.45 |  |  |  |  |  |  |  |
|  | 06 | 3 ± 0 | 1 ± 0 |  |  |  |  |  |  |  |
|  | 07 | 3 ± 0 | 1.25 ± 0.45 |  |  |  |  |  |  |  |
|  | 08 | 2 ± 0 | - |  |  |  |  |  |  |  |
|  | 09 | 2.33 ± 0.52 | - |  |  |  |  |  |  |  |
|  | 10 | 2 ± 0 | 1 ± 0 |  |  |  |  |  |  |  |
|  | 11 | 2.25 ± 0.45 | 1 ± 0 |  |  |  |  |  |  |  |
|  |  |  |  | [multiple comparison result]  06,09,17,21,23 > others | | | | |  |  |
|  | 12 | 2.35 ± 0.61 | 1.25 ± 0.45 |  |  |  |  |  |  |  |
|  | 13 | 2.17 ± 0.57 | 1.43 ± 0.51 |  |  |  |  |  |  |  |
|  | 17 | 2.36 ± 0.6 | 1 ± 0 |  |  |  |  |  |  |  |
|  | 20 | 2 ± 0 | - |  |  |  |  |  |  |  |
|  | 21 | 2.5 ± 0.55 | - |  |  |  |  |  |  |  |
|  | 23 | 2.33 ± 0.5 | - |  |  |  |  |  |  |  |
|  | 27 | 1.33 ± 0 | 1 ± 0 |  |  |  |  |  |  |  |
| Reposition | 03 | 1.86 ± 0.65 | - | 0.005* | | 0.169 | - | |  |  |
|  | 04 | 2.06 ± 0.64 | - |  |  |  |  |  |  |  |
|  | 06 | 2.33 ± 0 | - |  |  |  |  |  |  |  |
|  | 07 | 2.33 ± 0.43 | - |  |  |  |  |  |  |  |
|  | 10 | 2.5 ± 0.77 | - |  |  |  |  |  |  |  |
|  | 11 | 2.2 ± 0.7 | - |  |  |  |  |  |  |  |
|  | 12 | 2.19 ± 0.36 | - |  |  |  |  |  |  |  |
|  | 13 | 2.3 ± 0.41 | - |  |  |  |  |  |  |  |
|  | 14 | 3 ± 0 | - |  |  |  |  |  |  |  |
|  | 17 | 2.29 ± 0.65 | 1 ± 0 |  |  |  |  |  |  |  |
|  | 22 | 2.5 ± 0.58 | - |  |  |  |  |  |  |  |
|  | 27 | 2 ± 0 | - |  |  |  |  |  |  |  |
| Feeding | 04 | 1.9 ± 0.69 | 1 ± 0 | <.001* | | 0.931 | 0.111 | |  |  |
|  | 10 | 1 ± 0 | - |  |  |  |  |  |  |  |
|  | 11 | 2 ± 0 | - |  |  |  |  |  |  |  |
|  | 12 | 2 ± 0 | - |  |  |  |  |  |  |  |
|  | 13 | 1.9 ± 0.21 | 1 ± 0 |  |  |  |  |  |  |  |
|  | 17 | 1.65 ± 0.76 | 1.33 ± 0.5 |  |  |  |  |  |  |  |
|  | 18 | - | 1 ± 0 |  |  |  |  |  |  |  |
|  | 19 | - | 1 ± 0 |  | | | | |  |  |
|  | 22 | 3 ± 0 | 1 ± 0 |  |  |  |  |  |  |  |
|  | 23 | 2 ± 0.89 | 1 ± 0 |  |  |  |  |  |  |  |
|  | 25 | 2 ± 0 | - |  |  |  |  |  |  |  |
|  | 26 | 1.5 ± 0.71 | 2 ± 0 |  |  |  |  |  |  |  |
|  | 27 | - | 1.33 ± 0.52 |  |  |  |  |  |  |  |
| Toileting | 03 | 1.83 ± 0.38 | - | <.001* | 0.006* | | | 0.019* |  |  |
|  | 04 | 2.33 ± 0.66 | 1.5 ± 0.58 |  |  |  |  |  |  |  |
|  | 06 | 2 ± 0 | 1 ± 0 |  |  |  |  |  |  |  |
|  | 07 | 2 ± 0 | 2 ± 0 |  |  |  |  |  |  |  |
|  | 08 | 1.5 ± 0.58 | - |  |  |  |  |  |  |  |
|  | 09 | 3 ± 0 | - |  |  |  |  |  |  |  |
|  | 10 | 1.75 ± 0.45 | 1.5 ± 0.55 |  |  |  |  |  |  |  |
|  | 11 | 1.67 ± 0.5 | 1.33 ± 0.5 | [multiple comparison result]  04,09,15 >others | | | | |  |  |
|  | 12 | 2.14 ± 0.21 | 1 ± 0 |  |  |  |  |  |  |  |
|  | 13 | 2.22 ± 0.7 | 1 ± 0 |  |  |  |  |  |  |  |
|  | 15 | 3 ± 0 | - |  |  |  |  |  |  |  |
|  | 17 | 2.33 ± 0.69 | 1.22 ± 0.42 |  |  |  |  |  |  |  |
|  | 23 | 2 ± 0 | 2 ± 0 |  |  |  |  |  |  |  |
|  | 27 | 2 ± 0 | - |  |  |  |  |  |  |  |

Abbreviation: VAS = Visual analogue scale

Values are presented as mean ± standard deviation

^*^*p* < .05 calculated using a generalized linear model

- : All values are zero
